# 铁死亡的发生机制及其在肺癌中的研究进展

**DOI:** 10.3779/j.issn.1009-3419.2020.104.16

**Published:** 2020-09-20

**Authors:** 金铭 吴, 丽芳 马, 佳谊 王, 永霞 乔

**Affiliations:** 1 200025 上海，上海交通大学公共卫生学院 Public Health School, Shanghai Jiaotong University, Shanghai 200025, China; 2 200030 上海，上海市胸科医院，胸部肿瘤研究所 Shanghai Institute of Thoracic Tumors, Shanghai Chest Hospital, Shanghai 200030, China

**Keywords:** 肺肿瘤, 铁死亡, 激活剂, 分子机制, Lung cancer, Ferroptosis, Agonist, Molecular mechanism

## Abstract

铁死亡是近年来发现的一种铁依赖性的程序性细胞死亡，这种死亡是脂质过氧化物和活性氧堆积引起的。而肺癌作为病死率最高的恶性肿瘤之一，关于肺癌中铁死亡的研究甚少。本文中我们综述了肺癌中铁死亡受抑制的研究进展，从铁死亡发生的不同途径来解释肺癌中铁死亡受抑制现象。此外，诱导铁死亡治疗癌症越来越受到重视，本文中我们介绍了四种铁死亡激活剂以及铁死亡治疗肺癌的新展望，以期为肺癌治疗提供新的思路。

## Abstract (en)

Ferroptosis is a recently recognized form of regulated cell death caused by an iron-dependent accumulation of lipid reactive species. However, little research on ferroptosis and lung cancer, one of the most common tumors, has been carried out. This paper tries to review the research progress of ferroptotic suppression and explain it from the different ways of ferroptosis occurrence. Furthermore, as inducing ferroptosis to treat cancer gets more attention, we introduce four kinds of ferroptosis-inducing compounds and new prospects for lung cancer therapy to provide new ideas for lung cancer treatment.

铁死亡是近年来发现的一种区别于细胞凋亡和细胞坏死的程序性细胞死亡，这一过程的特点是依赖铁离子及活性氧（reactive oxygen species, ROS）诱导脂质过氧化物（lipid peroxides, LPO）堆积。磷脂中的多不饱和脂肪酸（polyunsaturated fatty acid, PUFA）、氧化还原活性铁以及LPO的修复缺陷是铁死亡的3个特征，它们决定了肿瘤细胞对铁死亡的敏感性^[1-3]^。肿瘤细胞比正常细胞更依赖铁来促进其高增殖率，这种现象被称为铁成瘾（iron addition）^[4]^。

肺癌是全球上最常见的恶性肿瘤之一，在2018年估计有210万新病例和近170万人死亡^[5]^，而肺癌发病率和死亡率在我国居所有肿瘤第一位^[6]^。肺癌病理类型主要有非小细胞肺癌（non-small cell lung cancer, NSCLC）和小细胞肺癌（small cell lung cancer, SCLC），前者又分为肺鳞癌（squamous cell carcinoma, SCC）、肺腺癌（adenocarcinoma, ADC）、大细胞肺癌等^[7]^，近年来肺癌病理类型以ADC的上升和SCC的下降为主要趋势^[8]^。

铁死亡的发现使得人们对于肿瘤疾病的发生发展有了新的认识。诱导铁死亡对于肿瘤治疗的重要性已经越来越受到重视，同时研究发现肿瘤细胞会产生铁死亡抵抗。在治疗肺癌的药物中，有一些已被证实可以诱导铁死亡，研究也发现肺癌细胞中存在铁死亡抑制的现象。那么，阐明铁死亡受抑的机制、诱导铁死亡发生对于治疗肺癌有着重要的临床意义。

## 1 铁死亡的发生机制

铁死亡发生的主要原因是细胞代谢方式产生改变，使细胞内LPO和ROS大量累积。在铁死亡过程中，主要有4条代谢通路发生改变。

### 1.1 谷胱甘肽（glutathione, GSH）合成通路受抑

细胞表面存在胱氨酸/谷氨酸转运体（cystine/glutamate antiporter, system Xc^-^）以维持氧化还原稳态，促使胱氨酸进入细胞，随后将其还原为半胱氨酸，将多余谷氨酸排出细胞，为细胞内GSH合成提供原料，同时GSH是谷胱甘肽过氧化物酶4（glutathione peroxidase 4, GPX4）降解LPO的必需反应底物^[9]^。当GSH合成通路受抑时，LPO积累，铁死亡发生。

### 1.2 铁元素大量累积

铁元素如何促进铁死亡尚不完全明确，如今公认的解释为二价铁离子可转移电子给胞内氧，使后者和脂质发生反应形成LPO^[10]^。Fe^2+^在微酸性条件下和肿瘤细胞内H_2_O_2_反应生成高活性的羟基自由基（·OH），即发生所谓的芬顿（Fenton）反应，·OH进而与细胞内的脂质体反应生成LPO^[11]^。

铁元素的吸收、消耗、储存以及周转都会对铁死亡的敏感性产生影响。转铁蛋白（transferrin, TRF）以及转铁蛋白受体1（transferrin receptor 1, TFR1）可将胞外铁元素转运入细胞^[12]^，多余的铁储存在铁蛋白中，各类铁蛋白可以通过维持铁稳态负性调节铁死亡过程。

### 1.3 脂质合成亢进

一些谷氨酰胺转运蛋白如SLC1A5存在于细胞表面，将细胞内萘乙酸排出细胞外以吸收外部谷氨酰胺。谷氨酰胺进一步合成谷氨酸、α-酮戊二酸，并最终产生脂质。如果脂质合成受刺激，它将提供形成LPO的原料，从而促进铁死亡^[12]^。

### 1.4 补偿GPX4缺失的酶催化系统

除了上述3种代谢通路调节铁死亡的发生，最新的一些研究鉴定出了独立于经典的GPX4通路的铁死亡抑制因子（ferroptosis suppressor protein 1, FSP1），并且发现FSP1的豆蔻酰化修饰对于抑制铁死亡的活性非常关键。

此外，其他细胞代谢机制例如甲羟戊酸、NADPH、硒元素以及聚胺代谢均可以影响铁死亡敏感性^[13, 14]^，因此铁死亡是一种与细胞代谢密切相关的细胞程序性死亡。

## 2 肺癌中铁死亡受抑

近年来，一些研究者发现，肺癌细胞中普遍存在铁死亡被抑制的现象。肺部组织较其他组织处于高氧浓度的环境中，这种特殊环境使得肺部肿瘤需要承受很大的氧化压力。因此肺癌细胞为了避免在转化过程中出现被易化和强化的铁死亡，采取了多种措施提高铁死亡的诱导阈值，从而导致铁死亡受抑，进而促进肺癌的发生发展。

### 2.1 GSH合成通路上调

Huang等^[15]^和Ji等^[16]^发现肺癌细胞通过上调System Xc^-^，直接靶向GSH，增加细胞抗氧化能力。SLC7A11（xCT）和SLC3A2是System Xc^-^的重要组成部分，其在肿瘤细胞系集中表达，表明这些细胞中的System Xc^-^活性可能更高。Huang等^[15]^利用寡核苷酸芯片发现，9株肺癌细胞株中，A549、HOP-62、NCI-H226、NCI-H322M和NCI-H460这5株细胞中SLC7A11呈高表达；其中A549中SLC7A11和SLC3A2表达量最高。Ji等^[16]^研究发现SLC7A11在NSCLC细胞的胞质膜中高表达，以促进气道上皮细胞的增殖和谷氨酰胺依赖性，并降低ROS的产生，抑制了铁死亡。

Lai等^[17]^则发现肺癌细胞通过直接上调GPX4的表达来抑制铁死亡，丝氨酸苏氨酸酪氨酸激酶1（serine threonine tyrosine kinase 1, STYK1）在NSCLC细胞SW900中高表达，进而促进GPX4的表达，促进了肺癌细胞的增殖，减弱了由铁死亡引起的多种线粒体异常，导致铁死亡在NSCLC中受到抑制。

### 2.2 铁元素减少

铁死亡是一种铁依赖的细胞死亡方式，铁代谢方面的改变也会抑制铁死亡。Kukulj等^[18]^发现，NSCLC患者肺癌组织中普遍高表达铁蛋白，血清铁蛋白表达增加，研究发现，血清铁蛋白越高，提示肿瘤患者远期预后越差。Ji^[19]^等发现铁蛋白大于300 mg/L的肺癌患者生存率明显降低。Shi等^[20]^发现铁蛋白水平在肺癌远处转移方面有统计学差异，低铁蛋白水平组的铂类药物化疗反应率也高于高水平组。这可能是因为，H-铁蛋白（铁蛋白H亚基）具有铁氧化酶活性，基于这个特性能够催化高毒性的二价铁转化为低毒性的三价铁，不稳定铁池（labile iron pool, LIP）水平降低，因此抑制了Fenton反应，抑制了铁死亡^[21]^。除了抗氧化外，异常表达的铁蛋白还可以通过免疫抑制、促进血管生成，促进细胞增殖等途径，促进肿瘤的发生发展^[22]^。

Alvarez^[23]^等发现肺癌组织和细胞系中铁硫簇合生物合成酶1（iron-sulfur cluster biosynthetic enzyme, NFS-1）普遍高表达，进一步实验发现NSF-1从半胱氨酸中收集更多的硫元素产生铁硫簇，细胞释放铁减少，明显缓解了高氧诱导的细胞铁死亡，动物实验中也发现NSF-1敲除后细胞成瘤时间明显延长，这表明为了克服高氧诱导的细胞铁死亡，肺癌发生过程中存在对NSF-1的阳性选择。

### 2.3 脂质合成抑制

铁死亡的特点之一是LPO大量累积，因此脂质合成受抑制也会抑制肺癌中的铁死亡。Jiang等^[24]^发现肺癌细胞通过淋巴样特异性解旋酶（lymphoid-specific helicase, LSH）调节脂质代谢来对抗铁死亡，LSH作为一种DNA甲基化修饰剂，通过激活脂质代谢相关基因，包括葡萄糖转运蛋白1（glucose transporter 1, GLUT1）、铁死亡相关基因固醇辅酶A去饱和酶1（sterol-CoA desaturase 1, SCD1）和脂肪酸去饱和酶2（fatty acid desaturase 2, FADS2），与WDR76蛋白（WD40蛋白家族成员）相互作用抑制铁死亡，研究观察到LSH过表达通过降低细胞内铁和ROS的水平来抑制铁死亡。这一机制也从表观遗传学角度解释了铁死亡受抑制现象。

### 2.4 补偿GPX4缺失的酶催化系统

除了传统的GPX4通路，最新的研究发现FSP1是独立于GPX4通路之外的铁死亡抑制因子。有研究^[25]^采用了合成致死CRISPR-Cas9的筛选方案，发现FSP1豆蔻酰化修饰后定位于细胞质膜，在质膜中，FSP1作为一种氧化还原酶，降低辅酶Q10（coenzyme Q10, CoQ10）水平，生成亲脂自由基捕获型抗氧化剂（radical-trapping antioxidant, RTA），阻止LPO的增殖，进一步发现FSP1在肺癌细胞的培养和小鼠肿瘤移植中介导抗铁死亡作用。在肺癌细胞中，当GPX4失活后，FSP1的存在能够维持肺癌细胞的生长。而Doll等^[26]^则通过在GPX4缺失的情况下进行筛选，找到能够补偿GPX4缺失引起铁死亡的FSP1蛋白。他们也发现在FSP1的N端存在经典的豆蔻酰化修饰的基序，会完全影响其抗铁死亡的能力。FSP1过表达的情况下，铁死亡被抑制。

### 2.5 其他途径

同样涉及到LSH，Wang等^[27]^的研究则首次揭示了长链非编码RNA（long noncoding RNA, LncRNA）作为竞争性内源RNA抑制肺癌细胞铁死亡的新机制，提示表观遗传修饰影响铁死亡的另一种途径：RNA测序发现LSH上调LncRNA LINC00336的表达，并在ADC临床样本中验证了LINC00336的异常高表达，LSH通过抑制p53活性而上调RNA结合蛋白ELAV1（ELAV-like RNA-binding protein 1, ELAVL1）表达，ELAVL1则通过与LINC00336结合而提高其稳定性，进而LINC00336作为竞争性内源RNA吸附微小RNA（microRNA, miR）6852并降低MIR6852活性，从而使miR6852靶基因胱硫醚β-合酶（cystathionine-β-synthase, CBS）表达上调，CBS是铁死亡的替代标志物，最终抑制铁死亡而促进肿瘤的发生发展。

肺癌中铁死亡受抑是个复杂的过程且相关分子机制尚不清楚，目前为止尚缺乏在临床标本水平较深入阐明铁死亡及相关代谢与肺癌间相关性的研究。而针对临床中ADC的比例越来越高，上述肺癌细胞系涵盖了ADC、SCC等NSCLC以及大细胞肺癌等，并没有特定研究说明ADC与铁死亡之间有独特的抑制通路。

现将全球主要针对肺癌中铁死亡受抑研究情况汇总于表 1中。

**1:** 肺癌中铁死亡受抑相关研究进展 Research progress on ferroptotic suppression in lung cancer

Researcher	Nationality	Related Pathway	Target	Conclusions/Results
Huang Y^[15]^	USA	GSH synthesis pathway	System Xc^-^	Over expression of SLC7A11 and SLC3A2 in lung cancer cells
Ji XM^[16]^	USA		System Xc^-^	Over expression of SLC7A11 in NSCLC cells
Lai Y^[17]^	China		GPX4	Over expression of GPX4 in NSCLC cells due to STYK1
Kukulj S^[18]^	Croatia	Iron metabolism pathway	Ferritin	Reduced LIP due to over expression of ferritin
Ji M^[19]^	China			
Alvarez SW^[23]^	USA		NSF-1	Reduced LIP due to over expression of NSF-1
Jiang YQ^[24]^	China	Lipid synthesis pathway	LSH	Activation of lipid metablic and ferroptotic genes due to over expression of LSH
Bersuker K^[25]^	USA	FSP1 synthesis pathway	FSP1	Halted propagation of LPO due to over expression of FSP1 with CRISPR-Cas9 screen
Doll S^[26]^	Germany			Halted propagation of LPO due to over expression of FSP1 with expression cloning approach
Wang M^[27]^	China	Epigenetic pathway	LSH	Over expression of transsulfuration marker CBS due to LSH
SCLC: small cell lung cancer; NSCLC: non-small cell lung cancer; LPO: lipid peroxides; GPX4: glutathione peroxidase 4; NFS-1: iron-sulfur cluster biosynthetic enzyme; LSH: lymphoid-specific helicase; CBS: cystathionine-*β*-synthase.				

## 3 诱导铁死亡与肺癌治疗

从认识到铁死亡是一种独特的调节性死亡模式，越来越多的研究表明铁死亡是抗癌疗法发展的新机遇^[28]^。铁死亡可由4类化合物引发，这些化合物称为铁死亡诱导剂（ferroptosis-inducing agents, FINs）^[28, 29]^。此外还有一些新型材料或药物诱导铁死亡的发生。

### 3.1 已有的临床药物

实际上，在已有的肺癌经典治疗中，一些治疗药物被揭示其抑癌机制与铁死亡有关。研究^[30]^发现顺铂是NSCLC A549细胞的铁死亡和凋亡的诱导因子，顺铂引起的还原型GSH的耗竭和GPX4的失活在其机制中起着重要作用，进一步发现顺铂与Ⅰ类FIN Erastin联合治疗对其抗肿瘤活性有显著的协同作用。而其他肺癌一线治疗药物，如替尼类药物、单抗药物、长春瑞滨、紫杉醇等药物，暂时未有详尽研究这些药物与铁死亡关系的实验。有参考价值的是乳腺癌药物拉帕替尼与抗焦虑药物西拉美新的组合通过降低TRF和铁蛋白的表达，增加LIP，诱导乳腺癌细胞铁死亡^[31]^。因此，一线肺癌治疗药物与铁死亡之间的关系尚需要大量研究来阐明。

### 3.2 理论上的FINs

#### 3.2.1 Ⅰ类FIN

Ⅰ类FIN（例如Erastin及其功能相关化合物）可抑制System Xc^-^，进而抑制下游半胱氨酸以及GSH的合成^[32]^，最终导致LPO累积和铁死亡发生。Erastin及其类似物哌嗪酮类erastin已被证实在宫颈癌、卵巢癌和纤维肉瘤的动物模型中抑制肿瘤生长^[28, 33, 34]^。

具体到诱导铁死亡治疗肺癌中，免疫抑制药物柳氮磺胺嘧啶（sulfasalazine, SASP）被发现显著降低了GSH水平，抑制了NCI-H69和LU6-SCLC组织异种移植物的生长^[35]^。抗癌药物索拉菲尼（sorafenib）也被发现在肺癌细胞系NCI-H460诱导了铁死亡^[36]^，这可能是由于索拉菲尼能够阻断System Xc^-^，抑制GSH的合成。

#### 3.2.2 Ⅱ类FIN

Ⅱ类FIN直接与GPX4交互并抑制其功能。Ras选择性致死性小分子3（ras-selective lethal small molecule 3, RSL3）与GPX4的亲核活性位点硒代胱氨酸共价相互作用，并抑制其酶促活性，导致其脂质修复功能丧失、LPO积累以及随后的细胞铁死亡^[3]^。尽管Ⅱ类FIN的药代动力学差^[37]^，但在未来可能具有癌症治疗的潜力。

#### 3.2.3 Ⅲ类FIN

Ⅲ类FIN如FIN56通过直接靶向并激活甲羟戊酸途径（mevalonate pathway）下游的、参与胆固醇合成的角鲨烯合成酶（squalene synthase, SQS）促进铁死亡，使得一些代谢物如脂溶性抗氧化剂CoQ10和Sec-tRNA受抑制^[38]^，间接耗竭或灭活GPX4。他汀类药物如西力伐他汀、辛伐他汀等可抑制甲羟戊酸途径的限速酶HMG CoA还原酶，通过消耗CoQ10抑制下游tRNA使细胞对铁死亡敏感^[39]^。

#### 3.2.4 Ⅳ类FIN

这一类物质主要增加LIP或氧化铁的水平，导致LPO积累，通常被称为非经典铁死亡诱导^[29]^。

血红素加氧酶-1（heme oxygenase-1, HMOX1）催化血红素降解为铁、胆绿素和一氧化碳，因而提高LIP的水平来增强铁死亡^[40, 41]^。用含铁化合物（如氯化铁、血红蛋白、血红素或硫酸亚铁铵）处理某些细胞会引发铁死亡^[40, 42-44]^。1, 2-二氧戊环（1, 2-dioxolane, FINO_2_）直接氧化铁，间接抑制GPX4，导致肿瘤细胞发生铁死亡^[45, 46]^。

可以看到，体外发现的可诱导铁死亡的药物可明显抑制肿瘤细胞的生长，尽管Ⅱ类、Ⅲ类、Ⅳ类FIN还未进行体内实验。这些铁死亡诱导小分子的优点可以预测到，它们很容易在体内代谢或清除，以防止低长期毒性。然而，由于肾脏的快速清除，药物靶向差，肿瘤部位的堆积较低^[40]^。此外，这些药物的副作用是明显的，因为正常细胞同时受到了损伤^[47]^。因此，稳定、持续地诱导肿瘤细胞发生铁死亡的药物还有待进一步研究。

### 3.3 铁死亡治疗新展望

#### 3.3.1 氧化铁纳米粒子（iron-oxide nanoparticles, IO NPs）

铁基材料是一种理想的肿瘤治疗方法，因为铁本身是产生ROS的Fenton反应的关键成分，这些材料可以通过被动和主动靶向聚集在肿瘤部位，然后释放亚铁或铁离子的酸性溶酶体，参与Fenton反应和诱导铁死亡杀死肿瘤细胞^[48]^，大大降低了铁死亡的不利影响。基于这些特性，人们开发了许多新的高效的方法，通过改变纳米材料的铁相关特性可以显著提高纳米材料的抗癌效率。

治疗缺铁性贫血的药物Ferumoxytol已被用于抑制早期乳腺癌和肺癌在肝、肺转移的生长^[49]^。顺铂负载的IO NPs诱导铁死亡，既增强了顺铂抗癌活性，同时最小化了全身毒性^[50]^。

#### 3.3.2 肿瘤免疫治疗

近年来肿瘤免疫治疗越来越受到重视，活化的淋巴细胞可以特异性识别肿瘤细胞并通过释放穿孔素和颗粒酶来诱导肿瘤细胞发生凋亡。研究^[51]^还发现肿瘤免疫治疗后，活化的CD8^+^ T细胞释放的γ-干扰素（interferon-γ, IFNγ）会下调SLC7A11和SLC3A2的表达，从而抑制肿瘤细胞对胱氨酸的摄取，增强肿瘤细胞的脂质过氧化和铁死亡，并且PD-L1抗体与胱氨酸降解酶偶联物cyst(e)inase联合治疗可以加强T细胞抗肿瘤效应。

#### 3.3.3 肿瘤放射治疗

实验观察发现电离辐射（ionizing radiation, IR）后，LPO增加而GSH含量降低^[52]^，并且能通过瞬态的水辐射分解和线粒体的变化产生氧自由基和ROS ^[53, 54]^，从而损坏核酸、蛋白质和脂质并引起细胞损伤乃至死亡，这强烈提示铁死亡在放疗的抗肿瘤效应中发挥了作用。而在最新的研究^[55]^发现，放疗后肿瘤细胞呈现典型的铁死亡形态学变化—线粒体浓缩，膜密度增高，线粒体脊减少，也即放疗可以通过产生大量ROS，并上调关键酶的表达，共同促进脂质过氧化，最终导致铁死亡；更为重要的是，在后续的实验中，用肺癌细胞系测试了Ⅰ类、Ⅱ类、Ⅲ类FINs的放射增敏作用，发现所有测试的FINs联合放疗都可以协同诱导脂质过氧化和铁死亡，并且明显增加肿瘤细胞对放疗的敏感性。

现将已有的与铁死亡相关的有临床应用潜能的药物和化合物汇总于表 2中。

**2:** 铁死亡诱导治疗相关的临床药物和化合物 Clinical drugs and compounds related to ferroptosis-inducing treatment

Classification		Drugs/Compounds	Target	Phenomena/Conclusion
Existing clinical drugs		Cisplatin^[30]^	GSH/GPX4	Combination therapy of cisplatin with erastin showed significant synergistic effect on their anti-tumor activity^[30]^
		Lapatinib^[31]^	LIP	Lapatinib and siramesine treatment induced ferroptosis in breast cancer cells^[31]^
FINs	Calss Ⅰ FIN	SASP^[35]^	System Xc-/GSH	SASP restrained growth of NCI-H69 and LU-SCLC tissue xenografts^[35]^
		Sorafenib^[36]^		Sorafenib induced ferroptosis in NCI-H460 cells^[36]^
	Class Ⅱ FIN	-	GPX4	Not suitable for *in vivo* use due to poor pharmacokinetic^[37]^
	Class Ⅲ FIN	-	SQS/GPX4	Cerivastatin and simvastatin inhibit mevalonate pathway enzyme, making cells sensitive to ferroptosis ^[39]^
	Class Ⅳ FIN	Iron compound^[40, 42-44]^	LIP	Iron compounds induced ferroptosis in cancer cells
		HMOX1^[40, 41]^		
		FINO2^[45, 46]^		
New prospects		IN OPs	LIP	Ferumoxytol has been used to inhibt the growth of lung cancer metastases^[49]^ IN OPs with cisplatin(Ⅳ) prodrugs enhanced anticancer activity but minimized systemic toxicity ^[50]^
		Immunotherapy	System Xc^-^	Immunotherapy-activated CD8^+^ T cells enhance lipid peroxidation in cancer cells^[51]^
		Radiation therapy	GSH/GPX4	IR induces ferroptosis in cancer cells and class Ⅰ, Ⅱ, Ⅲ FINs exhibit radiosensitization^[55]^
GSH: glutathione; SASP: sulfasalazine; IN OPs: iron-oxide nanoparticles; LIP: labile iron pool; SQS: squalene synthase.				

综上所述，铁死亡的发现，为肺癌的治疗开辟了许多新的路径。不仅现有的一些药物治疗和放射治疗被发现可以诱导铁死亡，更多的FINs被发现可以抑制肿瘤的生长，尽管在肺癌治疗上的研究并没有开展很多。如何发现更多的FINs并进行体内实验，如何诱导肺癌细胞发生铁死亡的同时，保护其他正常细胞免受损害，是未来亟需解决的问题。

## 4 结论和展望

铁死亡作为近年来发现的区别于细胞凋亡、坏死、自噬的一种新的铁依赖性程序性死亡，有着独特的发生和抵抗机制，其在肿瘤治疗上的重要性已经得到证实，越来越多的靶向铁死亡治疗也正在研究中。而肿瘤细胞中铁死亡受到抑制这一现象亦受到广泛关注，目前为止尚缺乏具体的研究探究其机制。肺癌作为最常见的恶性肿瘤之一，在我国的发病率、死亡率位于第一，但是肺癌中铁死亡抑制与铁死亡治疗方面的研究目前还接近于空白。本文综述了肺癌中铁死亡受抑制的研究进展，同时介绍了诱导铁死亡治疗肺癌的情况，并将开展研究试图明确肺癌中铁死亡受抑制的机制，以期为肺癌治疗提供新思路以及为肺癌患者的生存和死亡提供新的诊断和治疗方法。
